# Vector competence re-evaluation of reared *Glossina palpalis gambiensis* for transmission of *Trypanosoma congolense* and *Trypanosoma brucei brucei* isolates for an experimental event

**DOI:** 10.1186/s13071-025-07223-x

**Published:** 2026-02-07

**Authors:** Tindwendé Justin Yaméogo, Alain Boulangé, Wendemanegde Ernest Salou, Adrien Marie Gaston Belem, Marc Desquesnes, Sophie Ravel, Geoffrey Gimonneau

**Affiliations:** 1 Centre International de Recherche—Développement sur l’Elevage en zone Subhumide, Bobo-Dioulasso, Burkina Faso; 2 MARAH-DGSV-Direction de l’Entomologie et de la Lutte contre les Maladies Animales à vecteurs (DELMA), BP 1087, Bobo-Dioulasso 01, Burkina Faso; 3 CIRAD, UMR INTERTRYP, 34398 Montpellier, France; 4 INTERTRYP, Université de Montpellier, CIRAD, IRD, Montpellier, France; 5 Université Nazi Boni (UNB), 01 BP 1091, Bobo-Dioulasso 01, Burkina Faso; 6 CIRAD, ENVT, Toulouse, France

**Keywords:** Experimental infection, *Glossina* colony, Insectary, Vector competence

## Abstract

**Background:**

Tsetse flies (Diptera: Glossinidae) are vectors of human and animal trypanosomes. The *Glossina palpalis gambiensis* Burkina Faso (BKF) colony, established in 1972 and rejuvenated once in 1981, is a long-standing closed colony used extensively for research and vector control. While its performance and competitiveness for sterile insect technique (SIT) programs are regularly monitored, its vector competence (VC) data are outdated. This study aimed to update the VC data of this 47-year-old colony (from the onset of experiment in 2019) against *Trypanosoma congolense* and *T. brucei brucei* in laboratory conditions using trypanosome clone and tsetse fly individuals from the BKF colony.

**Methods:**

Vector competence was studied by infecting rats with *T. congolense* IL1180 and *T. b. brucei* BE8P2P2, on which tsetse flies received their first blood meal. Dissections were subsequently performed at different time intervals.

**Results:**

Following experimental infections with *T. congolense* IL1180, 10.58% (20/189) of *G. p. gambiensis* developed mature infections (trypanosomes in the proboscis), resulting in an average VC index of 0.106. For *T. b. brucei* BE8P2P2, 4.21% (11/261) of flies developed mature infections (trypanosomes in the salivary glands), yielding an average VC index of 0.042.

**Conclusions:**

The VC for *T. b. brucei* aligned with previous findings from 21 years ago, though a different trypanosome isolate was used at that time. However, using the same trypanosome strain, the observed competence for *T. congolense* was 4.8 times higher than previously reported. These results raise questions about the long-term effects of insectary rearing on VC, particularly in the absence of prolonged parasite exposure.

**Graphical abstract:**

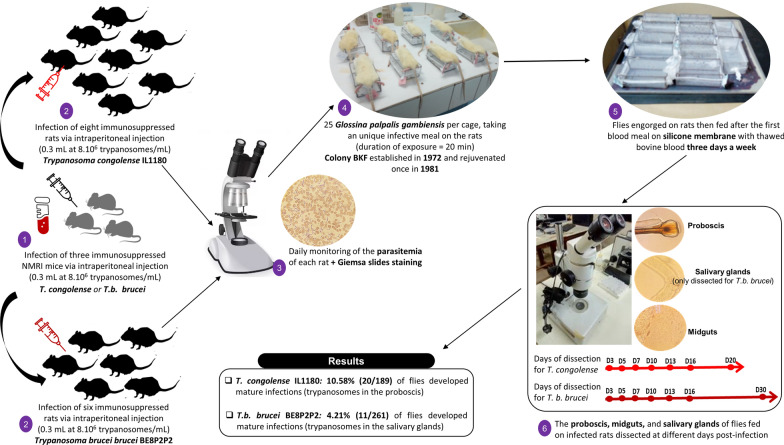

## Background

Tsetse flies (Diptera: Glossinidae) are the cyclical vectors of trypanosomes, the causative agents of animal African trypanosomosis (AAT) or nagana in animals, and human African trypanosomiasis (HAT), or sleeping sickness, in humans, two major scourges that impede development in Africa [1, 2]. Trypanosomes are extracellular flagellate protozoa, with the *T. brucei* species, *T. b. gambiense* and *T. b. rhodesiense*, mainly responsible for HAT. In addition, *T. b. brucei*, alongside *T. congolense* and *T. vivax*, causes AAT [3]. The presence of tsetse flies and AAT impairs the development of sustainable and productive agricultural systems in over 10 million km^2^ and is considered among the greatest constraints to livestock production in sub-Saharan Africa with an economic cost estimated at USD 4.75 billion per year [4, 5]. While HAT has been endemic in 38 sub-Saharan African countries, recent efforts to control HAT have drastically reduced the prevalence of the disease (new cases have been reduced by 97% in the last 20 years according to the World Health Organization (WHO), 2023). Moreover, the elimination as a public health problem of the *T. b. gambiense* HAT chronic form (g-HAT), has been achieved, and the WHO is now targeting the interruption of g-HAT transmission for 2030 [6]. This complex of diseases has an important impact on health and economic development in sub-Saharan Africa [7].

The potential transmission of a vector-borne disease has been used to predict risks of outbreaks, evaluate vector control strategies, and compare strains of a pathogen [8]. An accurate measure of this potential is critical, and often estimated by vectorial capacity. Originally devised by MacDonald [9], the vectorial capacity represents “the number of infections a specific vector population can distribute per case per day.” Another important parameter, necessary to accurately estimate the vectorial capacity, is the vector competence (VC), the ability of an arthropod to transmit an infectious agent following a previous exposure to that agent. A vector’s transmission capability is determined by its physiological ability to become infected with a pathogen, multiply the pathogen within its body, and ultimately transmit it to a new host. VC of tsetse for trypanosomes has been shown to be impacted by intrinsic and extrinsic (or abiotic) factors, such as biochemical conditions in the midgut of the fly, the presence of symbionts, the level of parasitemia in the host, as well as various environmental factors [10–12]. Estimates of VC can also be indicative of differences in vector susceptibility to trypanosomes strains. Indeed, several studies have shown that the VCs of tsetse vary according to the trypanosome subgenus and even subspecies [13–15].

Experimental evaluation of tsetse VC is conducted using a trypanosome strain originating from humans or animals, in conjunction with colony-reared tsetse individuals. Insect are reared in laboratories to produce colony populations from which a steady supply of the desired life-stage can be obtained for research. While these colonies are representative of the field populations from which they originate, long-term insectary rearing can influence the genetic background and fitness of the strain through selective pressure [16, 17]. Consequently, certain biological and/or physiological parameters may deviate from those observed in natural populations.

The Burkina Faso (BKF) colony of *Glossina palpalis gambiensis* was established in 1972 at Maisons-Alfort (France) using pupae and females collected from Guinguette (Bobo-Dioulasso, Burkina Faso) [18]. This colony, ever maintained at the Centre de coopération internationale en recherche agronomique pour le développement (CIRAD, Montpellier, France), has a long history of use in research. In 1975, the colony was transferred to the Centre de Recherche sur les Trypanosomoses Animales (CRTA) (later renamed Centre International de Recherche Développement de l’Elevage en zone Sub-humide (CIRDES)), using 5333 pupae. Subsequent introduction of wild flies occurred the same year with 1457 pupae collected near Bobo Dioulasso and in 1981 with flies from the “Mare aux hippopotames” located 70 km from Guinguette [19]. In 2016, approximately 54,000 adult flies from the CIRDES colony were transferred to the Insectarium de Bobo-Dioulasso (IBD) to establish a new mass-rearing colony [20]. At the time of our study in 2019, the IBD colony, which originated from the latter, was thus 47 years old. Assuming a mean generation time of 51 days at 25 °C (time to first ovulation: 8 days; pupal period: 32.9 days; interlarval period: 10.4 days) [19, 21, 22], this translates to approximately 336 generations of laboratory rearing.

Since the 1970s, the Burkina Faso-based BKF strain has been extensively studied, primarily within the context of biological control programs utilizing the sterile insect technique (SIT) [18, 23–25]. More recently, its performance has been evaluated in the context of a vector control program in Senegal [26]. While its VC has also been extensively investigated, particularly for *T. brucei gambiense*, for the France-based colony [27–30], studies on the Burkina Faso-based colony are limited. Previous studies were focused on the VC of this colony to *Trypanosoma (Nannomonas) congolense* IL1180 and *Trypanosoma (Trypanozoon) brucei brucei* EATRO 1125 [13, 14]. However, these data are outdated.

The VC can be influenced by various factors, including long-term insectary rearing, which can lead to changes in biological and physiological parameters probably linked to the absence of sustained parasite exposure and the effect of successive generations [31]. Therefore, this study aims to re-evaluate the VC of the 47-year-old BKF colony against *T. congolense* and *T. b. brucei*. While we could secure the same strain of *T. congolense* (IL1180), we did not get access to the *T. b. brucei* EATRO 1125 and resolved to utilize another strain present at CIRDES.

## Methods

To conduct this study, we used biological material such as tsetse flies, mice and rats as laboratory rodents, and two trypanosome strains.

Regarding tsetse flies, the species *G. p. gambiensis* was used. It was obtained from the Insectary of Bobo-Dioulasso—Anti-Tsetse Eradication Campaign (Insectarium de Bobo-Dioulasso, Campagne d’éradication de la mouche tsé-tsé et de la trypanosomose (IBD-CETT)). A total of 1390 2–3-day-old male and female flies from the closed IBD colony were used in this study.

For laboratory rodents, mice and rats of the Naval Medical Research Institute (NMRI), and Wistar strains from the CIRDES laboratory animal house, respectively, were used for the reactivation and multiplication of the trypanosome stabilates. A total of 6 NMRI mice (6 months old), and 14 Wistar rats (average age 7.5 months) were used.

Two strains of animal trypanosomes were used for experimental infections: (1) *T. congolense* Savannah IL1180, a clonal strain derived from an isolate obtained from a lion in Serengeti Park, Tanzania [32] and (2) *T. b. brucei* strain MSUS/CI/2013/BE8P2P2 isolated from a pig in 2013 in the Bonon endemic HAT focus in Côte d’Ivoire [33].

The two trypanosome stabilates were thawed at room temperature, and the parasite viability was checked by microscopy and used to infect three NMRI mice via intraperitoneal injection (0.3 mL at 8 × 10^6^ trypanosomes/mL) that had previously been immunosuppressed by intraperitoneal injection of 0.2 mL of freshly prepared cyclophosphamide (Endoxan^®^, 20 mg/mL). The parasitemia of each mouse was checked daily using the matching method [34]. When the parasitemia reached 6.4 × 10^7^ trypanosomes/mL, mice were euthanized by injection of 0.2 mL of ketamine hydrochloride at 50 mg/mL, followed by complete bleeding through cardiac puncture (in accordance with standard operating procedure used at the CIRDES laboratory to obtain sufficient inoculum to infect the rats). The blood (5 mL) was mixed with 0.1 mL of heparin to prevent coagulation and then diluted in phosphate-buffered saline glucose 1% (PSG 1%) to obtain a parasitemia of 8 × 10^6^ trypanosomes/mL. Rats previously immunosuppressed with 0.3 mL/rat cyclophosphamide (Endoxan^®^, 20 mg/mL), were infected by intraperitoneal injection with 0.3 mL of the suspension of 8 × 10^6^ trypanosomes/mL. Eight rats were infected with *T. congolense* IL1180 and six with *T. b. brucei* BE8P2P2. Parasitemia of each rat was then monitored daily, and Giemsa slide staining was also carried out to identify trypanosome forms.

As previous studies have demonstrated that tsetse flies are primarily infected during their first blood meal, teneral flies were only fed once on rats infected with trypanosomes when parasitaemia reached 6.4 × 10^7^–2.5 × 10^8^ trypanosomes/mL. For this, either male or female *G. p. gambiensis* flies were fed on the bellies of infected rats and anesthetized by intraperitoneal injection of 0.3 mL of ketamine in cages containing a maximum of 25 teneral flies for 20 min. Then, only the engorged flies were kept. Each rat was used to feed flies from two cages, one with males and one with females. A total of 790 teneral flies encompassing 396 males and 394 females were fed on rats infected with *T. congolense* IL1180 and 600 teneral flies comprising 300 males and 300 females on rats infected with *T. b. brucei* BE8P2P2. After this first and unique infective blood meal, all the engorged flies were subsequently fed 3 days a week on silicone membrane with thawed bovine blood. Indeed, tsetse are obligate blood feeders, requiring a meal every 2–5 days for survival [2].

To monitor the infection of trypanosomes and the establishment of the procyclic and metacyclic forms in the different organs of the flies, subsample specimens were dissected at specific time points during the experiment. Flies were randomly selected just before their next blood meal (i.e., 48 h after the last one) to reduce the presence of partially digested blood, and thus facilitate observation of trypanosomes in the midgut. The remaining flies were maintained through regular feedings to ensure their survival and allow continuation of the experimental timeline. Owing to their different tropism in the vector, metacyclic-form trypanosomes are sought in the salivary glands for *T. brucei* spp. and in the proboscis for *T. congolense* [35]. Particularly, the proboscis and midgut of flies fed on rats infected with *T. congolense* IL1180 were dissected on days 3, 5, 7, 10, 13, 16, and 20 [36]; whereas the proboscis, salivary glands, and midgut of flies fed on rats infected with *T. b. brucei* BE8P2P2 were dissected on days 3, 5, 7, 10, 13, 16, and 30 [37]. In total, 30 flies (15 males and 15 females) were dissected at each time point. However, depending on the number of flies surviving the experiment, the number of flies dissected varied for the last time points. For flies fed on *T. congolense* IL1180-infected rats, only 20 (10 males and 10 females) and 19 flies (11 males and 8 females) could be dissected on days 16 and 20, respectively. For flies fed on *T. b. brucei* BE8P2P2-infected rats, 81 flies (27 males and 54 females) were dissected on day 30.

At the end of the experiment, the collected data were analyzed. The blood intake rates of the flies were compared using a two-sample test for equality of proportions (prop.test function).

The VC index was calculated according to the formula of [38]: VC = *p* × *m* where “*p*” is the procyclic index, calculated as *n*′/*n*, where *n*′ is the number of flies that had trypanosome infection in the midgut and *n* the number of dissected flies.

The metacyclic index is represented as “*m*” and calculated as *n*″/*n*′, where *n*″ is the number of flies infected in the proboscis (for infections with *T. congolense*) or salivary glands (for infections with *T. b. brucei*) and *n*′ is the number of flies that had trypanosome infection in the midgut. All statistical analyses were performed with RStudio software version 3.6.4 (2019).

## Results

The results of feeding rate showed that a total 624 (out of 790) tsetse flies successfully fed on rats infected with *T. congolense* IL1180, and 548 (out of 600) flies fed on rats infected with *T. b. brucei* BE8P2P2, with a significantly (*P* < 0.001) higher blood feeding rate for the latter (Table 1). No difference in blood feeding rate was observed between males and females fed on rats infected with *T. congolense* IL1180 (*P* = 0.41), while blood feeding rate of females was significantly higher than of males (*P* = 0.002) for *T. b. brucei* BE8P2P2 (Table 1).

**Table 1:** Blood feeding results of male and female *G. p. gambiensis* fed on rats infected with *T. congolense* IL1180 or *T. b. brucei* BE8P2P2

Strain	Sex	Number of tested flies	Number of engorged flies	Feeding rate (%)
*T. congolense* IL1180	Female	396	306	77.27
	Male	394	318	80.71
	Total	790	624	78.98
*T. b. brucei* BE8P2P2	Female	300	285	95.00
	Male	300	263	87.66
	Total	600	548	91.33

According to the results regarding *T. congolense* IL1180, a total of 189 flies (96 males and 93 females) were dissected between day 3 and day 20 after the infective blood meal (Table 2). Procyclic trypanosomes were observed in the midgut from day 3 and up to day 20 in 57 flies (30.16%), and metacyclic trypanosomes were observed in the proboscis from day 10 up to day 20 in 20 flies (10.58%; Table 2).

**Table 2:** Infection indexes of *G. p. gambiensis* males and females infected with *T. congolense* IL1180

DPI	Sex	*N*	*n*′	*n*″	*p*	*m*	VC
D3	Male	15	7	0	0.466	0	0
	Female	15	11	0	0.733	0	0
	Total	30	18	0	0.600	0	0
D5	Male	15	2	0	0.133	0	0
	Female	15	6	0	0.400	0	0
	Total	30	8	0	0.266	0	0
D7	Male	15	1	0	0.066	0	0
	Female	15	0	0	0	0	0
	Total	30	1	0	0.033	0	0
D10	Male	15	5	1	0.333	0.200	0.067
	Female	15	1	1	0.066	1	0.067
	Total	30	6	2	0.200	0.333	0.066
D13	Male	15	3	2	0.200	0.666	0.133
	Female	15	6	3	0.400	0.500	0.200
	Total	30	9	5	0.300	0.555	0.166
D16	Male	10	4	3	0.400	0.750	0.300
	Female	10	3	2	0.300	0.666	0.199
	Total	20	7	5	0.350	0.714	0.249
D20	Male	11	4	4	0.363	1	0.363
	Female	8	4	4	0.500	1	0.500
	Total	19	8	8	0.421	1	0.421
Total	Male	96	26	10	0.270	0.384	0.104
	Female	93	31	10	0.333	0.322	0.107

*n* is the number of flies dissected; *n*′ represents the number of flies with procyclic trypomastigotes (midgut); *n*″ refers to the number of flies with metacyclic infection (proboscis); *p* = *n*′/*n*; *m* = *n*″/*n*′. The VC index was calculated according to the formula of [32]: VC = *p* × *m*, as described in the “Methods” section. DPI, day post infection

The overall procyclic index was 0.302, and no significant difference (*P* = 0.735) was observed between males and females (0.271 and 0.333 respectively, Table 2). The overall metacyclic index was 0.351, and no significant difference (*P* = 0.583) was observed between males and females (0.384 and 0.322 respectively, Table 2). The overall VC index was 0.106 and no significant difference (*P* = 0.872) was observed between males and females (0.104 and 0.107 respectively, Table 2).

For infections with *T. b. brucei* BE8P2P2, a total of 261 flies (117 males and 144 females) were dissected between day 3 and day 30 post-infective feed (Table 3). Procyclic trypanosomes were observed in the midgut from day 3 up to day 30 in 39 flies (14.9%), and metacyclic trypanosomes were observed in the salivary glands and proboscis only at day 30 in 11 flies (4.21%; Table 3).

**Table 3:** Infection indexes of *G. p. gambiensis* males and females infected with *T. b. brucei* BE8P2P2

DPI	Sex	*n*	*n*′	*n*″	*p*	*m*	VC
D3	Male	15	3	0	0.200	0	0
	Female	15	5	0	0.333	0	0
	Total	30	8	0	0.266	0	0
D5	Male	15	1	0	0.066	0	0
	Female	15	2	0	0.133	0	0
	Total	30	3	0	0.100	0	0
D7	Male	15	2	0	0.133	0	0
	Female	15	1	0	0.066	0	0
	Total	30	3	0	0.100	0	0
D10	Male	15	3	0	0.200	0	0
	Female	15	2	0	0.133	0	0
	Total	30	5	0	0.166	0	0
D13	Male	15	1	0	0.066	0	0
	Female	15	3	0	0.200	0	0
	Total	30	4	0	0.133	0	0
D16	Male	15	2	0	0.133	0	0
	Female	15	3	0	0.200	0	0
	Total	30	5	0	0.166	0	0
D30	Male	27	5	5	0.185	1	0.185
	Female	54	6	6	0.111	1	0.111
	Total	81	11	11	0.135	1	0.135
Total	Male	117	17	5	0.145	0.294	0.042
	Female	144	22	6	0.152	0.272	0.041

*n* is the number of flies dissected; *n*′ refers to the number of flies with procyclic trypomastigotes (midgut); *n*″ refers to number of flies with metacyclic infection (proboscis and salivary gland); *p* = *n*′/*n*; *m* = *n*″/*n*′. The VC index was calculated according to the formula of [32]: VC = *p* × *m*, as described in the “Methods” section. DPI, day post infection

The overall procyclic index was 0.149, and no significant difference (*P* = 1) was observed between males and females (0.145 and 0.152 respectively, Table 3). The overall metacyclic index was 0.282, and no significant difference (*P* = 0.492) was observed between males and females (0.294 and 0.272 respectively, Table 3). The overall VC index was 0.042 and no significant difference (*P* = 0.738) was observed between males and females (0.042 and 0.041 respectively, Table 3).

## Discussion

Evaluation of tsetse fly VC is crucial in understanding the epidemiology of human and animal trypanosomosis. These data are essential for refining epidemiological models and predicting transmission risk area, particularly given the ongoing global changes that are reshaping species distribution [39, 40]. *G. p. gambiensis*, a riparian species present throughout West Africa, is recognized as the primary vector for both human and animal trypanosomosis [41, 42]. Vector competence data are typically derived from laboratory experiments using individuals from colonies for standardization and logistical reasons. In spite of the different studies on the *G. p. gambiensis* BKF colony [13, 14, 27], to have continually available data about the VC of this now 47-year-old colony using two trypanosome strains proves to be necessary.

Although the *G. p. gambiensis* BKF colony has been maintained for decades on an artificial membrane feeding system, their rates of gorging on rats were high: 78.98% for flies fed on rats infected with *T. congolense* IL1180 and 91.33% for those infected with *T. b. brucei* BE8P2P2. Rates of the same order of magnitude were obtained by Kazadi et al. [13, 14] on guinea pigs and rats [43], demonstrating that decades of blood feeding on artificial membranes does not affect fly propensity to feed on live animals. The difference of rates of gorging obtained between the two strains could be explained by the fact that rats infected with these *Trypanosoma* strains may have undergone distinct metabolic modifications in the body, leading to the release of volatile substances that could have influenced the feeding behavior of the tsetse [44, 45].

On the basis of experimental infections of 2-day-old teneral flies with *T. congolense* IL1180, the VC observed was 0.106, which is 4.8 times higher than what was obtained by Kazadi et al. [14], who found a much lower VC of 0.022. Moloo and Kutuza [46], working on the same *G. p. gambiensis* colony from Burkina Faso 10 years before Kazadi et al. [14], found the same VC of 0.022 while using a distinct *T. congolense* strain from Nigeria. Despite this significant difference in VC, the establishment period of procyclic and metacyclic forms that we observed was the same timeframe observed by Kazadi et al. [13, 36] of 3 and 10 days, respectively. A study performed with flies from the *G. p. gambiensis* original France-based colony infected with *T. congolense* clone E325 [29] showed a similar intrinsic vector capacity as found in this study (VC = 0.138). However, in that experiment, the establishment period of procyclic and metacyclic forms was much longer (10 and 20 days, respectively) possibly due to difference of abiotic condition (*T* = 23 °C) and/or to the difference of trypanosoma strain used.

In contrast to the results obtained with *T. congolense*, experimental infection carried out with *T. b. brucei* BE8P2P2 showed that the VC and the establishment periods of procyclic and metacyclic forms are the same as those obtained by Kazadi et al. [13] 21 years ago (procyclic and metacyclic index of 0.08 and 0.04, respectively) possibly due to the substantial differences in the cyclic transmission of *Trypanosoma* in the same fly species despite different field isolates [28]. Metacyclic infection with *T. brucei* was only detected at day 30, a time at which the experiment ended. This limitation hinders a comprehensive assessment of the temporal dynamics of organ colonization in tsetse flies following trypanosome infection. Such a result may indicate either a reduced vector competence of the model used for *T. b. brucei*, or a strain-specific adaptation affecting its infectivity. Moreover, it is known that the parasitological method used (i.e., microscopy) did not allow for the detection of low parasite loads, highlighting that parasites were probably missed at earlier time points. Molecular analyses based on species-specific polymerase chain reaction (PCR) would have circumvented this limitation and should be included in future experimental infection studies.

The difference of VC observed for *T. congolense* between 1998 and now could be related to several factors that have been shown to influence tsetse susceptibility to infection [47], including: (1) fly age [48], (2) tsetse genetics [49], (3) tsetse symbionts [50], and (4) tsetse immune system [51]. Save for the age of the flies at the first blood meal, the other parameters cannot be controlled and have most likely evolved under insectary conditions, leading to the observed increase in VC for *T. congolense*. It is known that the older the teneral fly at the time of its first meal, the more likely it is to become infected [13]. In the present study, flies were 48–72-h-old, a day younger than in Kazadi et al. [13] at 80–96 h. Therefore, infection rates should have been lower or at least similar, whereas the VC was 4.8 times higher in the present study, meaning that other factors are affecting *G. p. gambiensis* VC. Multiple transmissions between rodents and flies in experimental infection studies between 1990 and 2019 may have led to genetic selection in favor of a strain more suited to infection by flies. On the fly side, genetic drift and selection in insectary are known factors that affect tsetse biology and physiology, but they also are symbionts [17]. Physiological factors such as lectin production in the midgut and hemolymph, involved in trypanosomes establishment in midgut and maturation [52, 53], may have changed during the last 21 years and flies have become more susceptible to *T. congolense*. However, this should also be the case for *T. b. brucei*, whose midgut establishment and maturation are also dependent on lectins [54]. The fact that we observed the same VC for the two strains of *T. b. brucei* at a 21-year interval may be artefactual, in the sense that, had we been able to use the same strain that was used in 1998, a difference of VC similar to the one detected with *T. congolense* may have emerged. Hence, contrary to the findings with *T. congolense*, no conclusive result can be drawn.

To summarize, our study highlights that the VC of a reared tsetse from a colony for the same trypanosome strain could change along decades and probably is no longer representative of the VC of wild tsetse flies. So, to study colony divergences over time, it would be interesting to compare infection parameters of the twin colonies such as the *G. p. gambiensis* colony present in France and Burkina Faso for the same trypanosome strains. Moreover, it would be valuable to conduct a comparative study by infecting tsetse flies from the same colony with several genetically distinct strains of the same trypanosome species (e.g., a laboratory strain and a recently isolated wild strain). Evaluating their vector competence through infection rates, metacyclic load, and infection kinetics using parasitological and molecular techniques would help determine whether the observed differences are truly attributable to strain-specific characteristics or methodological limitations.

## Conclusions

The vector competence (VC) of the 47-year-old *Glossina palpalis gambiensis* BKF colony was re-evaluated and found to have significantly increased for *Trypanosoma congolense* IL1180 compared with the results obtained during the last experimental infection conducted 21 years ago by Kazadi et al. [13, 14, 43]. In contrast, the VC for *T. b. brucei* remained unchanged, although a different strain was used, which complicates direct comparison and interpretation. These findings deserve further more in-depth and complementary research to highlight whether genetic and/or physiological changes may have occurred over time, increasing colony susceptibility. Therefore, studies using colony flies should be interpreted with caution when drawing general conclusions, especially regarding trypanosomes VC.

## Data Availability

The datasets used and/or analyzed during the current study are available from the corresponding author on reasonable request.

## Abbreviations

VC: Vector competence
*T. congolense* IL1180: *Trypanosoma congolense* IL1180
*T b. brucei* BE8P2P2: *Trypanosoma brucei brucei* BE8P2P2
